# Decreased Expression of the *Slc31a1* Gene and Cytoplasmic Relocalization of Membrane CTR1 Protein in Renal Epithelial Cells: A Potent Protective Mechanism against Copper Nephrotoxicity in a Mouse Model of Menkes Disease

**DOI:** 10.3390/ijms231911441

**Published:** 2022-09-28

**Authors:** Olga Haberkiewicz, Paweł Lipiński, Rafał R. Starzyński, Aneta Jończy, Patrycja Kurowska, Mateusz Ogórek, Aleksandra Bednarz, Sylwia Herman, Dawid Hatala, Paweł Grzmil, Zenon Rajfur, Zbigniew Baster, Małgorzata Lenartowicz

**Affiliations:** 1 Laboratory of Genetics and Evolution, Institute of Zoology and Biomedical Research, Jagiellonian University, Gronostajowa 9, 30-387 Kraków, Poland; 2 Department of Molecular Biology, Institute of Genetics and Animal Biotechnology, Polish Academy of Sciences, Jastrzebiec, Postepu 36A, 05-552 Magdalenka, Poland; 3 Laboratory of Physiology and Toxicology of Reproduction, Institute of Zoology and Biomedical Research, Jagiellonian University, Gronostajowa 9, 30-387 Krakow, Poland; 4 Department of Molecular and Interfacial Biophysics, Faculty of Physics, Astronomy and Applied Computer Science, Jagiellonian University, Łojasiewicza 11, 30-348 Kraków, Poland; 5 Cell and Developmental Biology Center, National Heart Lung and Blood Institute, National Institutes of Health, Bethesda, MD 20824-0105, USA

**Keywords:** CTR1 protein, *Slc31a1* gene, *Slc31a2* gene, copper, Menkes disease, kidney

## Abstract

Kidneys play an especial role in copper redistribution in the organism. The epithelial cells of proximal tubules perform the functions of both copper uptake from the primary urine and release to the blood. These cells are equipped on their apical and basal membrane with copper transporters CTR1 and ATP7A. *Mosaic* mutant mice displaying a functional dysfunction of ATP7A are an established model of Menkes disease. These mice exhibit systemic copper deficiency despite renal copper overload, enhanced by copper therapy, which is indispensable for their life span extension. The aim of this study was to analyze the expression of *Slc31a1* and *Slc31a2* genes (encoding CTR1/CTR2 proteins) and the cellular localization of the CTR1 protein in suckling, young and adult *mosaic* mutants. Our results indicate that in the kidney of both intact and copper-injected 14-day-old mutants showing high renal copper content, CTR1 mRNA level is not up-regulated compared to wild-type mice given a copper injection. The expression of the *Slc31a1* gene in 45-day-old mice is even reduced compared with intact wild-type animals. In suckling and young copper-injected mutants, the CTR1 protein is relocalized from the apical membrane to the cytoplasm of epithelial cells of proximal tubules, the process which prevents copper transport from the primary urine and, thus, protects cells against copper toxicity.

## 1. Introduction

Dietary copper predominantly absorbed in the duodenum and small intestine is the main source of this microelement in adult mammals [1,2,3]. Copper is transported from enterocytes to the blood, where it is bound to albumin, histidine or glutathione [3,4]. In these complexes, it is then transported via the portal vein into the liver. In hepatocytes, copper is bound to ceruloplasmin and again excreted to the circulation, and with blood transported to other organs and tissues [4,5,6]. Copper, when delivered to various cells, is utilized in metabolic processes mainly for the production of copper-dependent enzymes. Apart from the liver, the kidney plays a special role in the systemic homeostasis of copper. In the kidney, copper complexes are filtrated in glomeruli and copper is reabsorbed from the primary urine by the epithelial cells of the proximal renal tubules. Then, copper ions are transferred back to the circulation via the active form of Cu-ATP-ase, an ATP7A protein encoded by the *ATP7A* gene [6,7]. Due to the kidney-mediated copper recycling, only 2% of this microelement is removed from the body with the urine [3,4,6]. In the mammalian cells, copper uptake is mediated by transmembrane proteins CTR1 and CTR2 belonging to the CTR family, which are encoded by *Slc31a1* and *Slc31a2* genes, respectively [8,9]. CTR1 in the form of the functional homotrimer is located in the plasma membrane and forms a channel for Cu^+^ ions to pass through [10,11,12,13]. CTR2 is localized in the membranes of the vacuoles, vesicles, endosomes and lysosomes of mammalian cells [14,15,16]. CTR2 is characterized by a lower affinity for Cu ions compared to CTR1. The major role of CTR2 is to facilitate copper release from degraded cuproenzymes in lysosomes and to transport it back into the cytosol for reutilization [14,16]. However, little is known about the role and expression of CTR2 in the kidney. Regarding CTR1, previous studies have revealed that this copper transporter is expressed in the epithelial cells of the renal tubules [17,18,19] and contributes to the uptake of cuprous ions (Cu^+^) by tubular cells [12,15,19,20]. CTR1 is a high-affinity membrane copper transporter, basically found in the apical [19] and basolateral [17,18] membrane of the polarized epithelial cells of the renal tubules. However, upon high extracellular copper concentration, CTR1 is rapidly translocated to the cytoplasm and degraded [9,13,21]. The internalization of CTR1 can protect cells against copper toxicity, especially during disorders of copper metabolism caused by mutations in the *ATP7A* gene that are responsible for the ATP7A protein’s loss of activity. In addition, protection against the toxic effects of intracellular copper excess involves the relocation of the Cu-ATPases (ATP7A and ATP7B), which removes copper ions from the cells [3]. In humans, a lack of ATP7A activity caused by mutations in the X-linked *ATP7A* gene leads to Menkes disease (MD), a rare and lethal genetic disorder of the copper metabolism [22,23,24]. The ATP7A protein is involved in the delivery of copper to the secreted copper-dependent enzymes and in the export of excessive copper from the cells [3]. In patients with Menkes disease, the uptake of dietary copper by duodenal enterocytes is normal, but both intracellular traffic of this microelement and its transport to the blood across the basolateral membrane are disturbed. Similarly, in these patients, the loss of ATP7A activity results in the decreased recycling of copper from the primary urine to the blood, as under normal conditions ATP7A is known to participate in basolateral copper transport [6,25,26,27,28]. In consequence, copper is accumulated in the epithelium of the small intestine and in the renal cortex of the kidney, while its level in the serum and other tissues (including liver and brain) is extremely low [4,22,24,29]. In patients with Menkes disease, copper deficiency in the body leads to the reduction in cuproenzyme activity. This activity is essential for metabolic processes such as mitochondrial respiration (cytochrome c oxidase), the detoxification of free radicals (superoxide dismutase 1 and 3), pigment production (tyrosinase), neurotransmitter synthesis (peptidyl α-amidating monooxygenase) or connective tissue formation (lysyl oxidase) [3,4,28,30]. The clinical and pathologic features observed in Menkes patients clearly reflect the decreased activities of the above-mentioned enzymes. Boys with Menkes syndrome at the age of 1–2 months exhibit characteristic features such as depigmentation, hair that becomes tangled on the top of the head, pale skin, a face with micrognathia and pudgy cheeks [22]. However, they develop normally for 2–4 months, but in the course of further development they gradually lose some progressive skills, expand pathological symptoms and usually die by the age of 3–4 years [4,22,24,25,31]. The subcutaneous injection of copper–histidine complex soon after birth prevents neurological degeneration and improves the longevity of patients with Menkes disease [24,28,32,33,34]. Currently, this treatment is the only accepted form of copper therapy (NIH trial programme) [35]. Unfortunately, it leads to increased copper accumulation in the kidney [28].

In the present study, we used mice with a mutation in the *Atp7a* gene (*Atp7a^mo-ms^*) called *mosaic* mutants to analyze the expression and cellular distribution of the CTR1 protein in the kidney under pathological conditions. *Mosaic* mutants belong to the group of *mottled* mutations mice, which constitute an animal model of human Menkes disease, as they display similar disturbances of copper metabolism that are observed in patients with the mutation of the X-linked *Atp7a* gene [26,36,37,38]. Some of the *mottled* mutants develop a phenotype closely mimicking the Menkes syndrome and, thus, are an excellent model for the investigation of both the therapeutic and side effects of copper administration [2,27,37,39]. When injected therapeutically with copper, *mosaic* mutants survived significantly longer, but our previous results strongly indicated that such treatment leads to a massive copper accumulation in the kidney and has a detrimental effect on its structure and function [6,40,41,42,43].

Under physiological conditions, copper homeostasis in the kidney is sustained by the two main proteins: CTR1 and ATP7A, copper membrane importer and exporter, respectively. In mouse kidneys, ATP7A shows age-dependent intracellular localization [7]. However, no developmental studies of CTR1 transporter in the kidney have been reported. Although it is known that the expression of the *Slc31a1* gene at mRNA and protein levels can be influenced by intracellular and extracellular copper concentration [21,44], our knowledge of the regulation of the *Slc31a1* gene as well as CTR1 synthesis and degradation in the kidney is far from complete. Therefore, in the present study, we investigated the age-dependent expression of the *Slc31a1* gene at the mRNA and protein level in the kidney of mice under physiological and pathological conditions.

## 2. Results

### 2.1. Copper Concentration in the Liver, Kidney and Urine of Experimental Mice

The *mosaic* mutation leads to the lethality of the hemizygous (*ms/−*) males that die around the 16th day of postnatal life. Copper supplementation applied from the second day after birth extends the *mosaic* mutants’ life span and reduces some neurological symptoms such as tremors, ataxia or paralysis of the hind limbs [40,42,45]. However, in all age groups of the *mosaic* mutants, copper administration resulted in a significant increase in copper content in the kidney compared to both intact and CuCl_2_-injected w-t control mice (Table 1). Importantly, renal copper concentration in 14-day-old untreated mutants was significantly lower than in copper-treated ones. This clearly indicates that in mutants, copper therapy potentiates copper accumulation in the kidney. In contrast, copper concentration in the kidney of CuCl_2_-injected w-t mice was similar to that found in control individuals. In mammals, excess copper is predominantly stored in the liver [1,6]. Here, we show that in all age groups of *mosaic* mutants, even those supplemented with copper, the hepatic concentration of this microelement is significantly lower than in w-t controls (Table 1). The supplementation with CuCl_2_ of w-t mice resulted in significant hepatic copper accumulation only in suckling 14-day-old animals. More interesting is that 45-day-old w-t mice receiving copper supplementation up to day 44 after birth show a similar hepatic copper concentration as untreated mice (Table 1). We also measured copper concentration in the urine of experimental mice, except for 14-day-old untreated mutants, showing clear macroscopic signs of dehydration such as low body weight, loss of liquids due to diarrhoea, and an increased density of the plasma and erythrocyte acanthocytosis [45]. The obtained results reveal that in the two groups of w-t suckling mice, copper concentration in the urine is very low, while in mutants, copper administration resulted in a significant increase in copper concentration in the urine (Table 1). In 6-month-old mice, copper concentration in the urine was similar in all the investigated groups (Table 1).

### 2.2. Effect of Copper Supplementation on the Renal Expression of Slc31a1 and Slc3121 Genes at the mRNA and Protein Levels in 14-Day-Old Mosaic Mutant and W-T Mice

**Figure 1:**
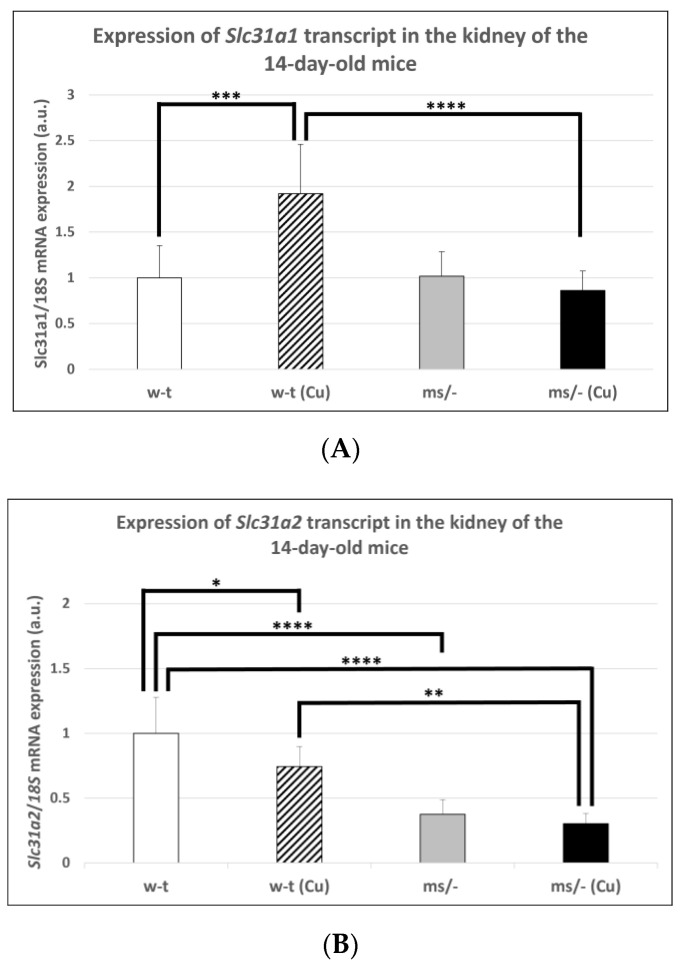
Expression of *Slc31a1* (**A**) and *Slc31a2* (**B**) genes in the kidney of the suckling (14-day-old), w-t and *mosaic* mutant mice. The histograms display mRNA levels in arbitrary units (means ± S.D., *n* = 5, (* *p* < 0.05, ** *p* < 0.01, *** *p* < 0.001, **** *p* < 0.0001).

The results of previous in vitro studies indicated that the expression of the *Slc31a1* gene encoding CTR1 is indirectly regulated by copper via the Sp1 transcription factor, which is down-regulated under copper-replete conditions but up-regulated under copper-depleted conditions [44,46,47]. However, little is known about the in vivo regulation of the *Slc31a1* gene expression in response to varying copper concentrations. Considering the elevated renal copper content in both mutant and w-t 14-day-old mice supplemented with CuCl_2_, we analyzed the expression of the *Slc31a1* gene in their kidney as well as in the kidney of untreated mutant and w-t controls. The results of the RT-q-PCR analysis show that the expression level of the *Slc31a1* gene was increased only in w-t mice injected with copper (Figure 1A). In mice from other groups (including copper-injected *mosaic* mutants), the expression of *Slc31a1* remained at a similar level (Figure 1A). In contrast, the expression level of the *Slc31a2* gene was significantly lower in the kidney of both copper administered to w-t and mutant mice and in non-injected mutants, as compared with w-t controls (Figure 1B). These results indicate that the expression of the *Slc31a2* gene can be down-regulated by copper, but the mechanism of this regulation is unknown.

To analyze the expression of CTR1 at the protein level in the kidney of experimental mice, we performed Western blotting on renal membrane extracts. Under conditions of non-denaturing SDS-PAGE, we revealed three electrophoretic migration positions of CTR1 at 25, 50, and 70 kDa, corresponding to the monomeric, dimeric and trimeric forms of the protein, respectively [19]. All three forms of the CTR1 protein were found in the kidney of mice from all investigated groups. Altogether, these results attest the presence of CTR1(trimeric form) in the membrane as well as in the cytoplasm (where the CTR1 protein removed from plasma membrane resides in vesicles in monomeric and dimeric form) [19] of the renal cells of analyzed mice.

### 2.3. Effect of Copper Supplementation on The CTR1 Protein Localization in the Kidney of 14-Day-Old Mosaic Mutant and W-T Mice

To further explore the expression and cellular localization of CTR1 in the kidney of examined mice we used the immunofluorescence method, keeping in mind that according to current knowledge the plasma membrane trimeric form of CTR1 is the only one active in copper transport [21,48].

**Figure 2:**
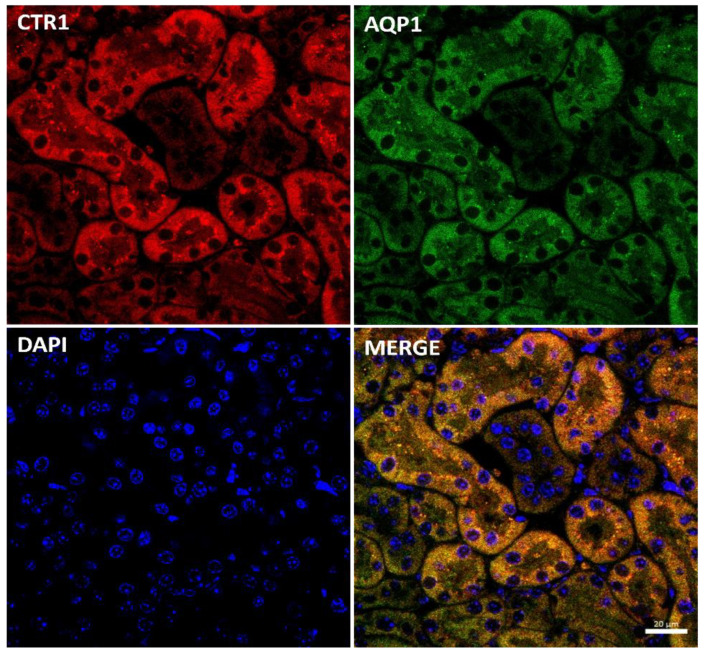
Co-localization of CTR1 (red channel) and aquaporin1 (AQP1, green channel), a proximal tubule marker in the kidneys of 14-day-old w-t mouse analyzed by confocal microscopy. Nuclei were counterstained with DAPI. Bar corresponds to 20 μm.

Immunostaining of the CTR1 protein in kidney sections of suckling mice was analyzed by confocal microscopy. To determine the precise localization of CTR1 in the cortical nephron, we performed double immunolocalization with aquaporin 1 (AQP1), a specific marker for proximal tubules [49,50] (Figure 2). A tight co-localization of CTR1 and AQP1 was found, indicating that in w-t mice, CTR1 is expressed in the proximal renal tubules, as shown in Figure 2. Similar localization of the CTR1 protein in the renal cortex was found in animals from other investigated groups of 14-day-old suckling mice.

**Figure 3:**
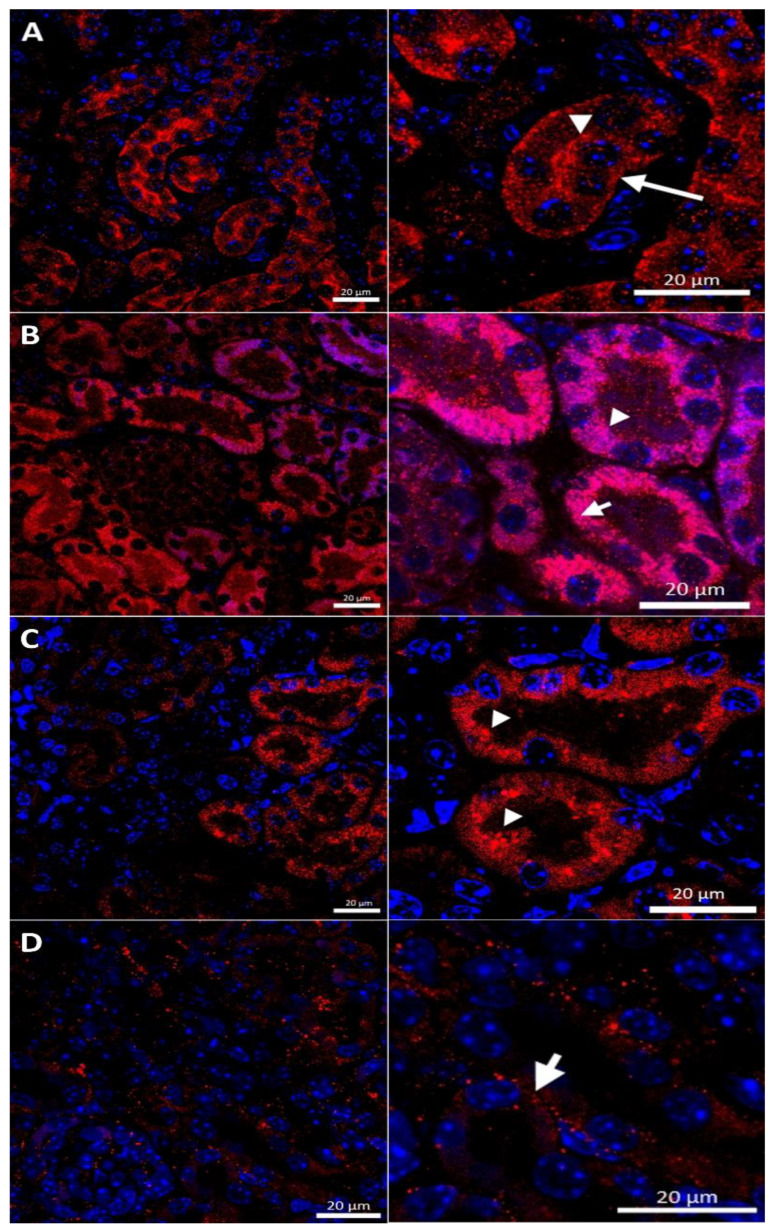
Immunofluorescent staining of the CTR1 protein (red, channel) analyzed by confocal microscopy in the kidney of the suckling (14-day-old). Right panels show digital enlargement of CTR1 staining visualized in left panels to reveal details of CTR1 cellular localization. This was performed using the ROI function of the ZEN programme. (**A**) Wild-type mouse, apical (arrow-head) and basolateral (long arrow) localization of CTR1 protein in the epithelial cells of the proximal renal tubules; (**B**) CuCl_2_-treated w-t mouse, visible apical (arrow-head) and cytoplasmic (little red dots (short arrow)) localization of the CTR1 protein in the epithelial cells of the proximal renal tubules; (**C**) untreated *mosaic* mutant, CTR1 protein exhibit apical (arrow-head) localization in the epithelial cells of the proximal renal tubules. (**D**) CuCl_2_-treated *mosaic* mutant, weak immunopositive signal observed in the form of the red dots, in the cytoplasm of the epithelial cells of the proximal renal tubules (short arrow) indicating for relocalization of CTR1 to the cytoplasm and degradation. Nuclei were counterstained with DAPI. Bars correspond to 20 μm.

In the renal cortex of w-t control mice, the CTR1 protein was found on the apical membrane (located from the side of the tubular lumen), and basolateral membrane (oriented away from the lumen of the tubule), of the proximal renal tubules (Figure 3A), where it participates in the processes of copper transport to the tubular cells. Apical localization is in line with the previous findings indicating that copper is reabsorbed from the primary urine mainly in this part of the nephron [7]. The basolateral expression of CTR1, visualized in Figure 3A, can indicate that copper is also transported to the tubular cells from the blood. It has been shown previously that the level of the CTR1 protein and its cellular localization is regulated by extracellular copper [21]. When cells are exposed to elevated copper levels, CTR1 is displaced from the plasma membrane and moves to the cell interior where CTR1 exhibits vesicular localization [21,48,51,52]. Not surprisingly, therefore, we found dual-membrane and cytoplasmic localization of the CTR1 protein in the cells of the renal tubules of w-t suckling mice supplemented with copper [w-t (Cu)]. We noticed that copper treatment results in the partial delocalization of the CTR1 protein from the plasma membrane and in the appearance of the intensive immunopositive signal in the cytoplasm (visible as little immunopositive dots), although in some tubules the CTR1 protein was still localized on the apical plasma membrane (Figure 3B). We have not found an expression of the CTR1 protein in the basal membrane of the epithelial cells of the renal tubules of copper-injected w-t mice (Figure 3B). In the kidney of suckling *mosaic* mutants, we observed staining of the CTR1 protein on the apical membrane of the epithelial cells of the renal tubules. Moreover, we noticed that on the apical membrane, the immunopositive signal attesting CTR1 expression was more intensive than that in control mice (Figure 3C). The effect of copper therapy on the cellular localization of the CTR1 transporter was strongly visible in the kidney of *mosaic* mutants. In the renal cortex of these mice, only in a few tubules was CTR1 was present on the apical membrane. Further, the extremely weak immunopositive signal was found in the cytoplasm, shown as little immunopositive dots in the epithelial cells of the renal tubules (Figure 3D). In copper-treated mutants, we have found that CTR1 is not present on the basal membrane of the epithelial cells of the renal tubules, which suggests a dramatic effect of copper on the CTR1 protein disappearance from this localization. Overall, both membrane and cytoplasmic weak CTR1 immunopositive signals strongly suggest the intensive degradation of the CTR1 protein in the cortex of copper-injected *mosaic* mutants.

### 2.4. Effect of the Long-Term Copper Supplementation on the Renal Expression of the Slc31a1 and Slc31a2 Genes at the mRNA Level in 45-Day-Old Mosaic Mutant and W-T Mice

**Figure 4:**
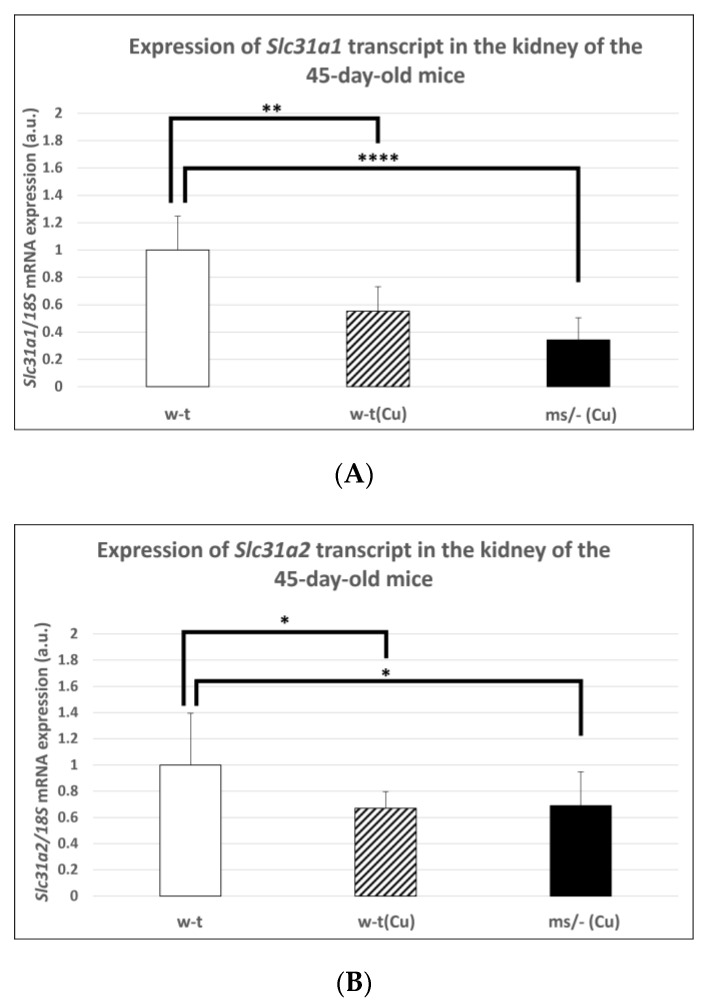
Decreased expression of the *Slc31a1* (**A**) and *Slc3a2* (**B**) genes in the kidney of 45-day-old copper-treated w-t and *mosaic* mutant mice. The histograms display mRNA levels in arbitrary units (means ± S.D., *n* = 5, (* *p* < 0.05, ** *p* < 0.01, **** *p* < 0.0001).

In subsequent experiments performed on mice of more than 14-days-old, we used animals from three groups because, as mentioned, the *mosaic* mutants non-supplemented with copper die by the age of 16 days. Extended copper supplementation (injections with CuCl_2_ from day 2 up to day 44 after birth) resulted in a significantly decreased expression level of the *Slc31a1* gene in the kidney of both w-t and mutant 45-day-old mice (Figure 4A). However, it should be noted that massive copper accumulation, which is supposed to decrease the expression of the *Slc31a1* gene, was only reported in copper-supplemented 45-day-old *mosaic* mutants (Table 1). The analysis of the expression of the *Slc31a2* gene also revealed that in both copper-treated w-t and *mosaic* mutant mice, the CTR2 mRNA level was significantly decreased in comparison with w-t controls (Figure 4B).

### 2.5. Effect of the Long-Term Copper Supplementation on the CTR1 Protein Localization in the Kidney of 45-Day-Old Mosaic Mutant and W-T Mice

**Figure 5:**
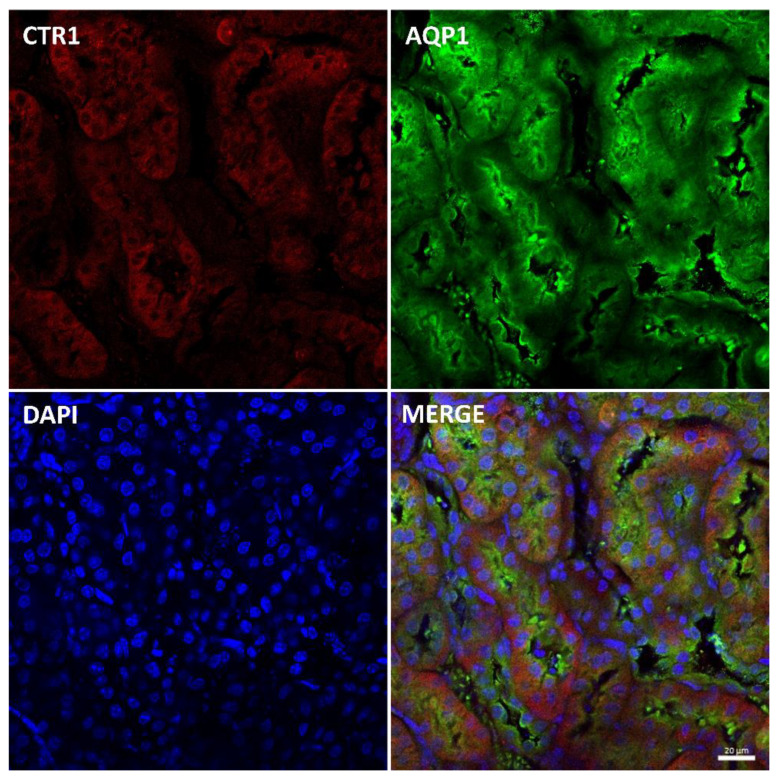
Immunofluorescent staining of CTR1 protein (red, channel) and aquaporin 1 (AQP1), a proximal tubule marker (green channel), in the kidneys of 45-day-old w-t mouse analyzed by confocal microscopy. Localization of both CTR1 and AQP1 in the epithelial cells of the proximal renal tubules. Nuclei were counterstained with DAPI. Bars correspond to 20 μm.

To analyze the localization of CTR1 in the cortical nephron of 45-day-old mice, we performed double immunolocalization with aquaporin 1 (AQP1), a specific marker for proximal tubules [49,50] (Figure 5). The localization of CTR1 and AQP1 indicates that in w-t mice, CTR1 is expressed in the proximal renal tubules, as shown in Figure 5. A similar localization of CTR1 protein in the renal cortex was detected in animals from all investigated groups of 45-day-old mice.

**Figure 6:**
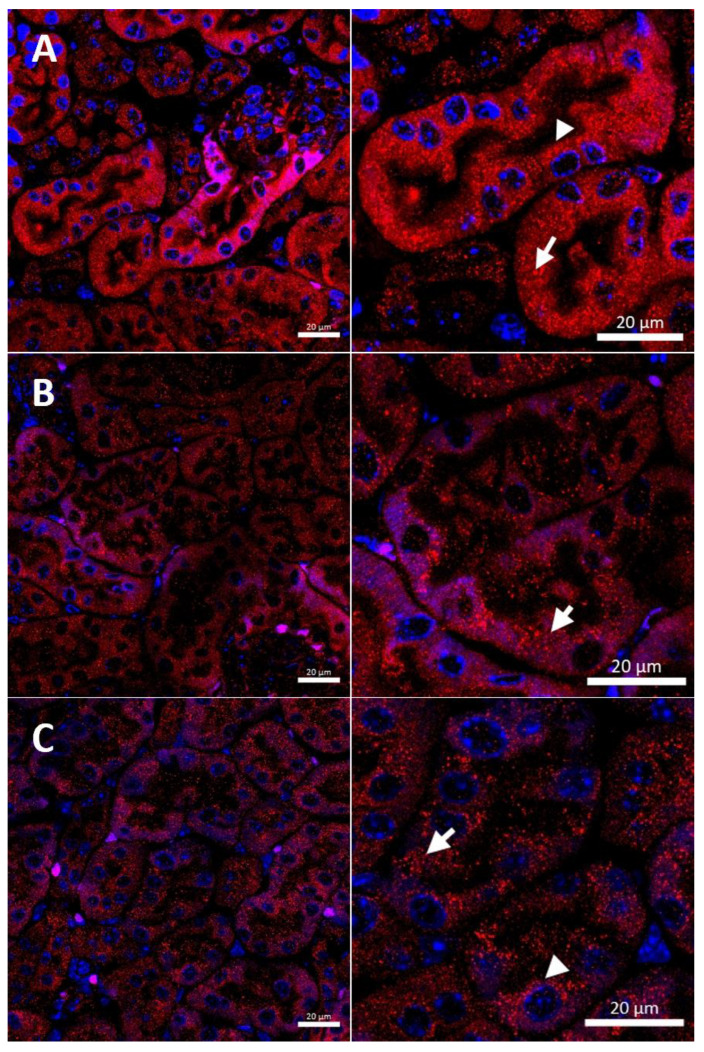
Immunofluorescent staining of CTR1 protein (red channel) analyzed by confocal microscopy in the kidney of the young (45-day-old). Right panels show digital enlargement of CTR1 staining visualized in left panels to reveal details of CTR1 cellular localization. This was performed using the ROI function of the ZEN programme: (**A**) W-t mouse, apical (arrow-head) and cytoplasmic (short arrow) localization (red dots) of CTR1 protein in the epithelial cells of the proximal renal tubules. (**B**) Cupric chloride-administered w-t mouse, cytoplasmic localization of CTR1 protein visible in the form of the red dots (short arrow) in the epithelial cells of the proximal renal tubules. (**C**) Cupric chloride-treated *mosaic* mutant. Cytoplasmic localization of the CTR1 protein (in the form of the red dots, short arrow) in the epithelial cells of the proximal renal tubules. Nuclei were counterstained with DAPI. Bars correspond to 20 μm.

In w-t control males, we observed the expression of CTR1 in the cytoplasm and weak immunopositive signal in the apical membrane of some renal tubules, pointing to the expression of CTR1 in the renal cortex (Figure 6A). In the renal cortex of copper-administered w-t mice, CTR1 was found to be present in the cells of the renal tubules with cytoplasmic, vesicular localization observed as immunopositive dots (Figure 6B). This indicates that when cells are exposed to the elevated copper concentration, CTR1 is displaced from the plasma membrane, which probably represents a protective mechanism against copper toxicity. In the renal cortex of copper-treated *mosaic* mutants, a weak immunopositive CTR1 signal was visible in both the apical membrane and the cytoplasm (immunopositive dots) of the cells of the renal tubules (Figure 6C).

### 2.6. Effect of the Long-Term Copper Supplementation on the Renal Expression of The Slc31a1 and Slc31a2 Genes at the mRNA Level in 6-Month-Old Mosaic Mutant and W-T Mice

**Figure 7:**
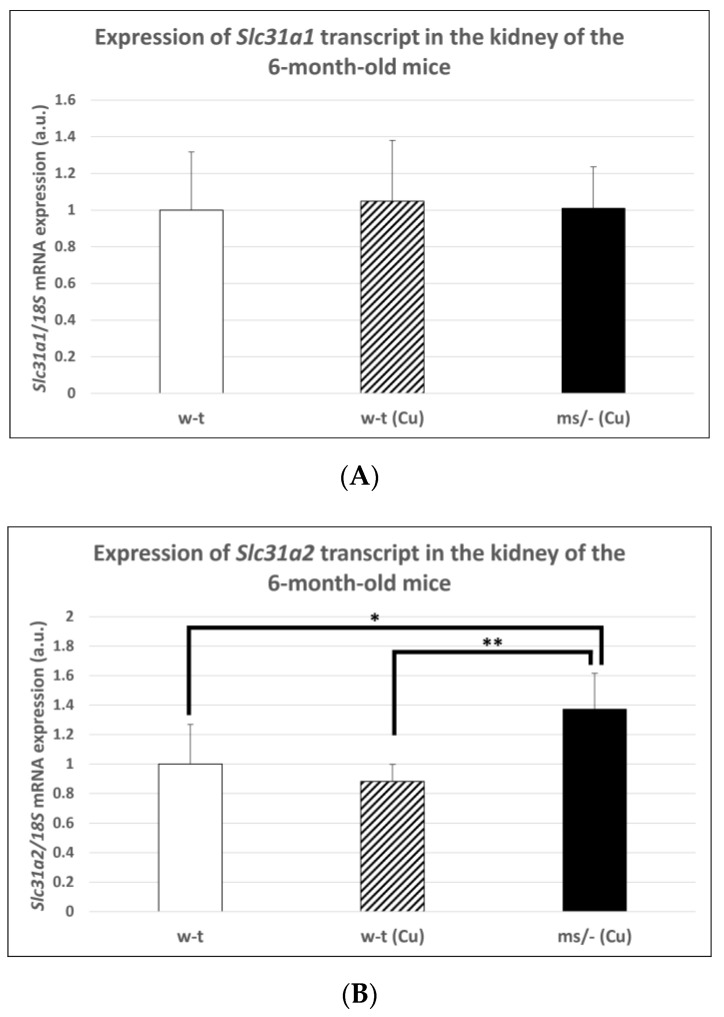
Expression of *Slc31a1* (**A**) and *Slc31a2* (**B**) genes in the kidney of adult (6-month-old), w-t and *mosaic* mutant mice. The histograms display mRNA levels in arbitrary units (means ± S.D., *n* = 5, (* *p* < 0.05, ** *p* < 0.01).

The copper treatment of w-t mice and *mosaic* mutants was stopped on day 44 after birth and mice (including non-treated w-t controls) being bred up to the age of 6 months. The reason for this was to investigate the long-term effect of copper therapy after its completion on *Slc31a1* and *Slc31a2* genes expression in the kidney of w-t and *mosaic* mutants. No significant differences in CTR1 mRNA abundance in the kidney were found between adult individuals from three experimental groups (Figure 7A). In contrast, we found that the expression of the *Slc31a2* gene in the kidney of the *mosaic* mutants is increased compared with mice from both w-t groups (Figure 7B).

### 2.7. Effect of the Long-Term Copper Supplementation on the CTR1 Protein Localization in the Kidney of 6-Month-Old W-T and Mosaic Mutant Mice

**Figure 8:**
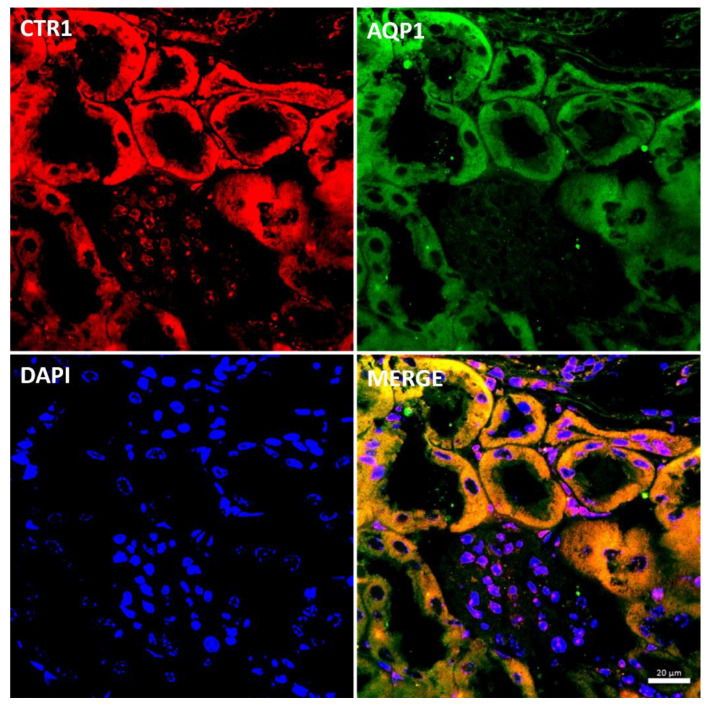
Colocalization CTR1 (red channel) and aquaporin1 (AQP1), a proximal tubule marker (green channel) in the kidneys of adult (6-month-old) w-t mouse analyzed by confocal microscopy. Nuclei were counterstained with DAPI. Bars correspond to 20 μm.

In the renal cortex of adult mice, localization of the CTR1 protein in the proximal renal tubules was confirmed by co-localization of the CTR1 protein with AQP, a specific marker for proximal tubules, and shown in Figure 8. Similar localization of the CTR1 protein in the renal cortex was found in animals from other investigated groups.

**Figure 9:**
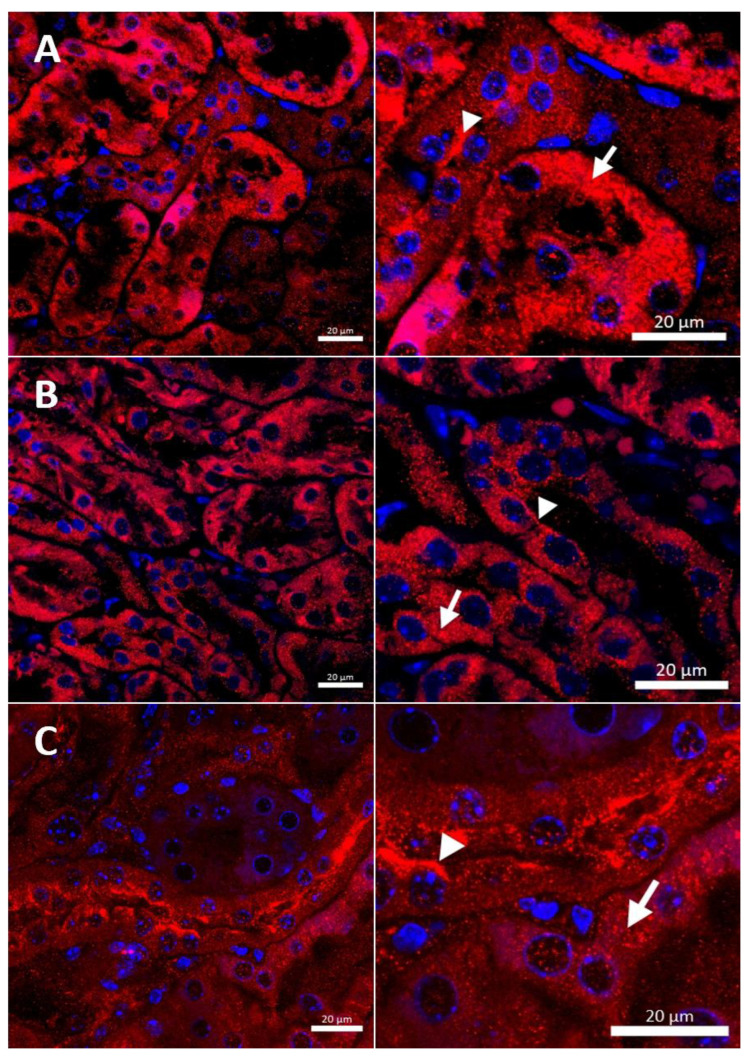
Immunofluorescent staining of CTR1 protein (red, channel) analyzed by confocal microscopy in the kidney of the adult (6-month-old). Right panels show digital enlargement of CTR1 staining visualized in left panels to reveal details of CTR1 cellular localization. This was performed using the ROI function of the ZEN programme: (**A**) W-t mouse, both apical (arrow-head) and cytoplasmic (visible as numerous red dots) (short arrow) localization of CTR1 protein in the epithelial cells of the proximal renal tubules. (**B**) Cupric chloride-administered w-t mouse, apical (arrow-head) and cytoplasmic (red dots) (short arrow) localization of CTR1 protein in the epithelial cells of the proximal renal tubules. (**C**) Cupric chloride-treated *mosaic* mutant, strong immunopositive signal indicating for CTR1 protein expression detected in the apical membrane (arrow-head) and in the cytoplasm (red dots) of the epithelial cells of the proximal renal tubules. Expression of CTR1 protein was also observed in the cytoplasm (short arrow) of the epithelial cells of the proximal renal tubules. Nuclei were counterstained with DAPI. Bars correspond to 20 μm.

In the renal cortex of w-t control mice, the immunopositive signal was found in the renal tubules and the expression of the CTR1 protein was localized in the cytoplasm. A weak signal was also detected in the apical membrane of the epithelial cells (Figure 9A). A similar expression pattern of CTR1 protein was noticed in the renal cortex of copper-injected w-t mice (Figure 9B). In contrast, in *mosaic* mutants, the CTR1 protein was mainly identified in the apical membrane of the epithelial cells of the renal tubules. A weak immunopositive signal of the CTR1 protein was also observed in the cytoplasm (Figure 9C).

## 3. Discussion

In mammals, the renal copper content is among the highest in the body because the kidney plays a pivotal role in copper redistribution in the organism [7]. In the kidney, copper is filtrated from the blood to the primary urine; next, in the nephron it is reabsorbed by the cells of the proximal tubules and transferred back to the blood. Both copper entry and exit into/from the epithelial cells of proximal tubules are tightly regulated and involve molecular mechanisms operating on the apical and basolateral cellular surfaces, respectively. The results of previous studies [17,19] and our present data indicate that copper uptake by the renal epithelial cells is, in a large part, mediated by a high-affinity Cu^+^ transporter, the CTR1 protein. On the other hand, copper export to the blood requires the activity of ATP7A copper transporting ATPase [3,7,26]. In the cells, the ATP7A protein exhibits perinuclear localization and is bound to the trans Golgi membrane (and sometimes vesicles) and attached to the plasma membrane. The intracellular localization of ATP7A depends on copper concentration in the cells; when copper concentration increased (for example, during the copper therapy), ATP7A bound the excess copper and transported it to the plasma membrane via vesicles that bud off from the trans Golgi network, releasing copper into the blood fluid [3,7,26]. A lack of activity of the ATP7A protein leads to copper accumulation in the kidney of the *mottled* mice [6,53,54,55] and patients with Menkes disease [56,57,58,59]. Moreover, therapeutic copper administration results in copper accumulation in the kidney of both patients and *mottled* mice [2,4,6,28,57,59,60,61]. Paradoxically, little is known about copper uptake in the kidney under pathological conditions caused by a mutation in the *ATP7A/Atp7a* gene. In the present study, we analyzed the expression of the CTR1 protein in the kidney of untreated and copper-treated *mosaic* mutant mice—an animal model of Menkes disease. We also analyzed the expression *Slc31a2* gene encoding for the second copper transporter—CTR2. Mouse *Slc31a2* gene is ubiquitously expressed in all tissues, but its highest mRNA levels were found in the heart, liver, kidney, testis and lower levels in muscle and brain [9]. In mammalian cells, the CTR2 protein localizes to intracellular vesicular compartments including endosomes and lysosomes, where its function is to mobilize vesicular Cu stores into the cytoplasm [9,62]. In contrast to CTR1, CTR2 is a low affinity copper transporter which is probably caused by the lack of His- and Met-rich motifs that bind Cu with high affinity to the ectodomain of the full-length CTR1 protein [62]. All these data indicate that in physiological conditions, CTR2 does not participate in cellular copper import, and thus, CTR1 remains the main copper importer in mammalian cells [9]. Therefore, in the present study, our analyses were focused on the expression and intracellular localization of the CTR1 protein in kidney cells.

Our present and previous [6] results indicate that copper administration leads to a significant increase in copper concentration in the kidney of suckling, young and adult *mosaic* mutants devoid of the ATP7A activity. Interestingly, despite continuous copper administration, its content in the liver of *mosaic* mutants from all investigated age groups was significantly lower than in w-t mice. The reason for this is that, in mutants, copper was trapped in the kidney and partially removed with the urine, as shown in suckling mice. The administration of CuCl_2_ (cupric chloride) to suckling, 14-day-old w-t mice resulted in hepatic copper accumulation. It is worth noting that the liver is the primary organ of copper deposition during the prenatal [1] and probably also early neonatal life. Interestingly, despite the long-time copper supplementation, copper content in the liver of 45-day-old w-t mice was similar to untreated controls. Moreover, at the age of 6 months, the hepatic copper level was similar in w-t control and CuCl_2_-injected mice. This is because in the liver of adult w-t mice copper is bound to the ATP7B protein and, in this complex, it is removed into the bile, or, in the form of Cu-ceruloplasmin complex, it is excreted to the blood [63]. With blood, copper is transported to the kidney and in the process of glomerular filtration passes to the primary urine. Glomerular filtrate formed in the renal corpuscle passes into the renal proximal convoluted tubules where the CTR1-mediated reabsorption of copper cations takes place. In the kidney of both w-t and mutant mice from all age groups, we detected CTR1 protein in the cells of the renal proximal tubules, and this cellular localization was attested by the co-localization of CTR1 with the aquaporin 1 protein, a specific marker of the renal proximal tubules [50]. The apical localization of the CTR1 protein can be connected with copper reabsorption from the primary urine, as it has been already postulated by Kuo et al., 2006 [19]. Next, in w-t mice copper is pumped back from the cells to the bloodstream by ATP7A, while in mutants it is trapped within the cells. The basal localization of the CTR1 protein in the cells of the renal tubules was previously described by Zimnicka et al., 2007 [17]. We suggest that the basal expression of CTR1 may be related to the transport of copper ions from the blood back to the cells. In the context of the basal localization of CTR1, it is worth noting that this CTR1 localization in the cells of the renal tubules contributes to the uptake of cisplatin, an anti-cancer drug, and leads to its accumulation in the cells and acute renal failure in patients during chemotherapy [18,46,64].

It is known that in mammalian cells, the expression of the *Slc31a1* gene (encoding for CTR1 protein) is regulated by copper concentration via the Sp1 transcription factor [44,46,47,64] interacting with the three GC boxes located at the *SLC31A1* promoter [44,47]. The expression of the Sp1 transcription factor is down- and up-regulated under copper overload conditions and copper deficiency, respectively [44,46,64]. Surprisingly, despite renal copper accumulation, we did not observe a decreased expression of the *Slc31a1* gene in the kidney of the 14-day-old mutants. Moreover, in the kidney of the 14-day-old w-t copper-injected mice, the expression of the *Slc31a1* gene was even up-regulated. The reason for this regulation remains unknown and needs further investigation. In contrast, we found that the expression of the *Slc31a2* gene in the kidney of suckling w-t copper-injected mice and both groups of *mosaic* mutant mice was decreased. This observation suggests that the expression of the *Slc31a2* in the kidney is down-regulated by an elevated concentration of intracellular copper. The immunohistochemical analysis of CTR1 localization showed that in the renal cortex of the kidney of suckling 14-day-old control w-t mice, CTR1 was located on both the apical and basal membrane of the epithelial cells of the renal tubules, which is indicative of intensive processes of copper reabsorption from the primary urine, as well as copper uptake from the blood by the epithelial cells of the cortical renal tubules.

In 14-day-old (suckling) w-t mice, the transport of copper across the epithelial cells of renal tubules from the primary urine to the blood seems to be very intensive, as attested by: (i) the predominant basolateral localization of ATP7A [7]; (ii) the strong expression of CTR1 on the apical membrane of these cells (shown in this study). Clear apical localization of the CTR1 protein in the epithelial cells of the proximal tubules of untreated *mosaic* mutants of the same age confirms that copper in these mice is also intensively reabsorbed from the primary urine by CTR1. However, the transport of copper to the blood is disturbed due to the malfunction of the ATP7A copper exporter, which is mainly localized in the cytoplasmic compartment [26].

In contrast to untreated w-t and mutant mice, in copper-administered animals, CTR1 exhibited intracellular localization in the renal cortex. Moreover, in copper-treated mutants, high copper concentration probably leads to partial degradation of the CTR1 protein. This is in line with previous reports showing that in cells exposed to elevated copper levels, CTR1 is aimed at copper-dependent endocytosis [21,48,51] and, thus, copper entry rate is lowered, and cells are protected against copper toxicity [48]. This relocalization and degradation of CTR1 in copper-treated mutants may lead to a decrease in copper reabsorption from the urine and consequently, as shown here, to the copper excretion with the urine. Although this copper release from the organism can protect kidney tissue from copper toxicity, on the other hand, it can reduce the effectiveness of copper therapy applied to these mice.

It is well established that copper therapy beginning from the second and lasting up to the 44th day of life reduces the lethality of *mosaic* mutation [6,40,42], although it leads to copper accumulation in the kidney [6,27,40,41]. In 45-day-old w-t and mutant copper-treated mice, the expression of the *Slc31a1* and *Slc31a2* genes was down-regulated compared to respective controls. The immunohistochemical localization of the CTR1 protein in the renal cortex of the kidney of 45-day-old mice revealed that in untreated w-t animals, CTR1 was located both in the cytoplasm and on the apical membrane of the epithelial cells of the renal tubules. The apical localization of CTR1 confirms that in 45-day-old mice copper is still absorbed from the primary urine [19], but this process is probably not as intense as in suckling mice because in 45- in contrast to 14-day-old animals, dietary copper is normally absorbed in the small intestine and, thus, covers a significant part of the systemic copper needs. Interestingly, the analysis of the localization of the ATP7A protein in the cortical tubules in the kidney of 8-week-old mice revealed that ATP7A is present in both perinuclear and vesicular compartments, and a few tubules exhibit staining close to the basolateral membrane [7]. This observation confirms that copper reabsorbed from the urine via CTR1 is transported to the blood by ATP7A. Different localization of the ATP7A protein copper administration to w-t males resulted in CTR1 protein internalization in the cells of the renal cortical tubules. We suggest that this delocalization of CTR1 protects renal cells against copper overload. Interestingly, in contrast to 14-day-old w-t copper-injected mice, 45-day-old copper-treated w-t animals did not show neither hepatic copper accumulation, nor a removal of copper excess with the urine (the latter according to the results of the AAS analysis). To avoid a toxic accumulation of copper, vertebrates developed a mechanism that allows the release of copper excess from the organism through ATP7B—the Cu-transporting ATPase [63]. It is likely that in young copper-overloaded w-t mice, copper excess is transported to the biliary surface of hepatocytes in the complex with the ATP7B protein, and then copper is released into the biliary space; in the end, the copper would be removed from the organism with faeces [3]. In 45-day-old *mosaic* mutants, due to the lack of ATP7A activity, copper accumulation in the cells of the cortical renal tubules induces the relocalization of CTR1 to the cytoplasm and its partial degradation. This mechanism can, to a certain extent, protect kidney cells from copper toxicity. However, long-time copper exposure leads to numerous pathological changes in kidney structure, especially in the copper-treated *mosaic* mutants [6]. Copper therapy applied from the second up to the 44th day after birth significantly extends the lifespan of the mutants, and some of them achieve longevity. However, according to our present and previous results [6,40], during their life, they continuously accumulate copper in the kidney, but at the age of 6 months, copper concentration in the plasma [40] and liver is lower than in control mice. In the group of 6-month-old mice, we did not find differences in the expression level of the *Slc31a1* gene in the kidney, but renal expression of the *Slc31a2* gene was higher in mutants. We can speculate that 6-month-old mutants that finished copper therapy on day 44 after birth develop a systemic copper deficiency, which in turn, up-regulates the expression of the *Slc31a2* gene. The results of the previous studies point to CTR2 as a vacuolar copper exporter that mobilizes copper from the vacuolar lumen into the cytosol under conditions of its extracellular scarcity [62,65]. The results of our immunohistochemical analysis revealed that in the kidney of 6-month-old mutants, the CTR1 protein was localized in the apical membrane of the renal proximal tubules, whereas in both groups of w-t mice it was mainly found in the cytoplasm of the epithelial cells of the renal tubule. This may suggest that in mutants suffering from copper deficiency, this microelement is still intensively reabsorbed from the primary urine, but due to the lack of activity of the ATP7A protein, copper is trapped in the epithelial cells of the renal tubules. The resulting massive copper accumulation causes numerous pathological changes in the kidney of 6-month-old *mosaic* mutant mice, including big lesions in the renal cortex, damage to the renal corpuscle, necrosis of the renal glomeruli and renal tubules [6,53]. Our results reveal that at the age of 6 months, *mosaic* mutants (injected with copper up to day 44 after birth) remain copper-deficient because, due to the lack of ATP7A activity, they do not absorb copper in the small intestine and rapidly deplete their hepatic copper stores. Thus, in the kidney of copper-deficient mutants, the CTR1 protein is localized in the apical membrane of the epithelial cells of renal tubules, allowing copper reabsorption from the primary urine. However, the lack of activity of ATP7A blocks copper transport to the blood. Meanwhile, in both groups of w-t mice, CTR1 is mainly localized in the cytoplasm, but in some cells of the epithelial cells of the renal tubules it exhibits membrane localization. This is in accordance with previous findings that in the kidney of 20-week-old mice, ATP7A is located almost entirely in the perinuclear region [7]. This indicates that in adult mice, the process of copper reabsorption and transport to the blood from the urine is not so intensive as in suckling and young individuals.

**Figure 10:**
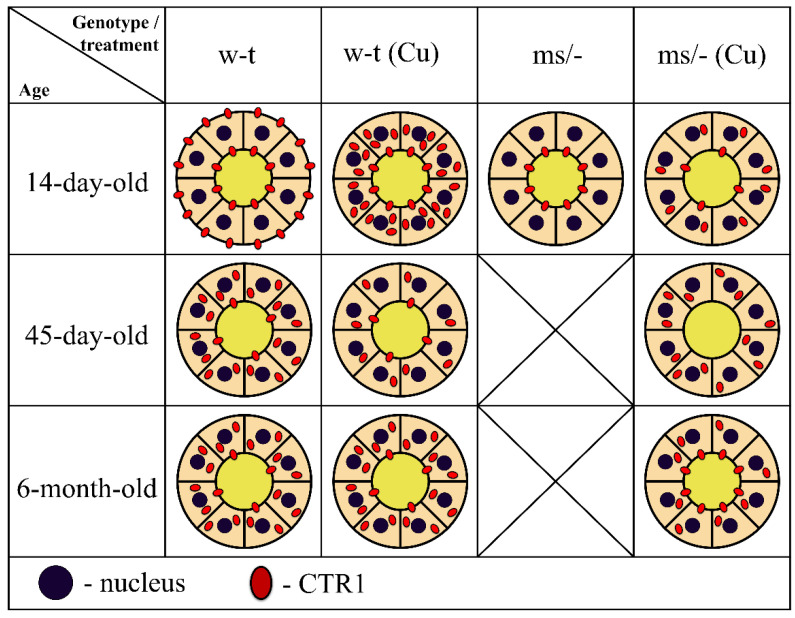
Schematic illustration of the CTR1 protein expression and distribution in epithelial cells of the renal proximal tubules in untreated and copper-treated w-t and *mosaic* mutant mice. Diagrams represent schematic structure of the proximal renal tubule, where inner and outer circles represent apical and basolateral membranes of the epithelial cells of the proximal tubule, respectively. Tubular lumen filled with yellow indicates primary urine. Membrane and/or cytoplasm localization and approximate amount of CTR1 protein (showed as red dots) was determined based on the results of the immunofluorescence analysis.

To summarize our results, in Figure 10 we provide a concise and illustrative scheme of CTR1 expression and distribution in the epithelial cells of the renal proximal tubules in untreated and copper-treated w-t and *mosaic* mutant mice with regard to developmental aspects.

In conclusion, our results show that in the kidney, under physiological conditions, copper reabsorption from the primary urine is regulated by the CTR1 protein, which is present on the apical membrane epithelial cells of the proximal tubules. Importantly, the flow of copper from these cells to the blood also depends on the activity of the ATP7A transporter, which is localized on the basolateral membrane. In pathological conditions caused by the dysfunction of ATP7A and manifested by intracellular copper overload, as in the case of *mosaic* mutants, the CTR1 protein is re-localized from the membrane to the cytoplasm, and degraded, thereby protecting kidney cells against copper toxicity. This phenomenon is strongly enhanced upon copper therapy applied to *mosaic* mutants with the purpose of extending their lifespan.

## 4. Materials and Methods

### 4.1. Animals and Copper Therapy

The mice used in the experiments were bred in the Department of Genetics and Evolution, Jagiellonian University, and derived from a closed-outbred colony. In the present study, we used *mosaic* mutant males—which exhibit a lethal phenotype at about day 16 after birth—and age-matched wild-type (w-t) males. *Mosaic* missense mutation (*Atp7a^mo-ms^*) consists of G to C nucleotide exchange at position 3009 of the cDNA, in the exon 15 of the *Atp7a* gene, resulting in an arginine to proline substitution at position 978 in the 6th highly conserved transmembrane domain of the ATP7A protein [26]. *Mosaic* mutant males (*ms/−*) were obtained by mating heterozygous *ms/+* females with normal (*+/*−) males. In our previous studies, we showed that copper treatment significantly prolongs *mosaic* mutants’ lifespan and partially reduces the pathological effects of the *mosaic* mutation [6,40,42]. Copper therapy applied to mutant males consisted of subcutaneous injections of 50 μL of 0.01% CuCl_2_ (Sigma-Aldrich, Saint Louis, MO, USA) solution every 2 days from day 2 to day 44 after birth. The total amount of copper administered per animal during this period was 100 μg. In the present study, we used males of three ages: 14-day-old (suckling), 46-day-old (young) and 6-month-old (adult). Suckling males were allotted to 4 experimental groups: copper-treated *mosaic* [*ms/−(*Cu)] and wild-genotype [w-t (Cu)] males and respective untreated controls: *ms/−* and w-t. As untreated *mosaic* mutant males die around 16 days after birth, our experiment on 45-day-old and 6-month-old mice included only 3 experimental groups: w-t, w-t (Cu) and *ms/−*(Cu). All mice were housed at a constant temperature (22 °C) under artificial light (12-h photoperiod) and fed a standard Labofeed diet (Motycz, Poland). The experiments were performed in accordance with Polish legal requirements under the licence of the First Local Ethical Committee on Animal Testing at the Jagiellonian University in Krakow (permission number: 85/2012).

### 4.2. Tissue Sample Collection

The animals were sacrificed by cervical dislocation and the kidney and liver were excised from the mice following a laparotomy. Tissues were immediately fixed or frozen in liquid nitrogen and stored at −80 °C prior to the molecular analysis.

### 4.3. Measurement of the Total Copper Content in Tissues

The copper content in the kidney and liver samples obtained from the experimental male mice was measured by atomic absorption spectrophotometry. The samples were weighed and digested in 2 mL of boiling Suprapur-grade nitric acid (Merck, Darmstadt, Germany). After cooling to room temperature (RT), each sample was suspended in 10 mL of deionized water. The reference material samples were prepared in a similar manner. The copper concentration was measured using the graphite furnace AAS technique (AAnalyst 800, Perkin-Elmer, Perkin-Elmer, Waltham, MA, USA). Three samples of nitric acid were used as blanks. In addition, three samples of standard reference material, Cu = 189 ± 4 mg/kg^−1^, were analyzed for the normalization of the obtained data.

### 4.4. Real-Time Quantitative RT-PCR

Total RNA was isolated from the kidneys of the experimental animals using an RNA isolation kit (Macherey-Nagel, Dueren, Germany). The RNA was treated with DNAse-I (Macherey-Nagel, Dueren, Germany) and reverse transcribed using a High-Capacity cDNA Reverse Transcription Kit (Applied Biosystems, Foster City, CA, USA). Specific fragments of the *Slc31a1* gene were then PCR amplified from this cDNA using the specific primers (forward primer 5′-GACAACATTACCATGCCACCTCACCA-3′ and reverse primer 5′-GTAAAAACACTGCCCACGAAGGCTCCA-3′. To amplify specific fragments of the *Slc31a2* gene, we used the following primers: forward primer 5′-CTCTTTGATTTCTGGAGGGTCCACAG-3′ and reverse primer 5′-TAGAATCCTGGTCTGGTCCCAAGATG-3′. To evaluate the relative expression level compared to *18S* (forward primer 5′-CTGAGAAACGGCTACCACATC-3′ and reverse primer 5′-CGCTCCCAAGATCCAACTAC-3′), the Delta-Delta Ct method was used. A real-time PCR was performed with the SYBR Green quantitative PCR kit (Applied Biosystems, Foster City, CA, USA) using a Step One thermocycler (Applied Biosystems, Foster City, CA, USA).

### 4.5. Immunofluorescence (IF) and Confocal Analysis of Kidney Sections

After sacrificing the mice, the kidneys were immediately excised and fixed in 4% paraformaldehyde (Sigma-Aldrich, Saint Louis, MO, USA) in phosphate-buffered saline (PBS) (Sigma-Aldrich, Saint Louis, MO, USA) at 4 °C for 24 h. After washing 3 times for 30 min in PBS, both tissues were successively soaked in 12.5 and 25% sucrose (Merck, Darmstadt, Germany) for 1.5 and 12 h, respectively at 4 °C. The tissues were then embedded in the Tissue-Tek compound, frozen in liquid nitrogen and sectioned into 20-µm slices using a cryostat (Shandon, UK). The sections were washed in PBS and permeabilized by bathing in PBS/0.1% Triton X-100 (Sigma-Aldrich, Saint Louis, MO, USA) for 10 min. Non-specific antibody binding was blocked by incubating the tissue sections in PBS/3% BSA (Merck, Darmstadt, Germany) for 1.5 h. For CTR1 detection in the kidneys, the sections were incubated at RT with primary rabbit polyclonal anti-CTR1 antibody (Novus Biologicals, Littleton, CO, USA) diluted 1:100 in PBS/3% BSA. The sections were then washed 3 times with PBS and incubated with Cy3 (indocarbocyanine)-conjugated goat anti-rabbit antibody (Jackson Immunoresearch, West Grove, PA, USA) diluted 1:500 in PBS/3% BSA. Finally, the sections were washed 3 times for 10 min in PBS at RT and mounted using Vectashield with 4′,6-diamidine-2-phenylindole (DAPI; Vector Labs, Newark, CA, USA). As a negative control, some sections were prepared without incubating with a primary antibody. IF was analyzed with a Zeiss LSM 710 Meta confocal microscope (Carl Zeiss, Jena, Germany) using the 60× objective. The presence of CTR1 in the epithelial cells of the proximal renal tubules was determined by double immunofluorescence localization of the investigated proteins and the proximal tubule marker aquaporin-1 (AQP-1) [49]. In order to distinguish the different proteins in this experiment, the secondary antibodies were conjugated with different fluorochromes: Cy3 goat anti-rabbit antibody (Jackson Immunoresearch, West Grove, PA, USA) for CTR1 and Alexa488 (Jackson Immunoresearch, West Grove, PA, USA) for marker AQP-1. For the immunolocalization of CTR1 and AQP-1, the standard IF protocol was performed using mixtures of the required primary and secondary antibodies. For the immunolocalization of CTR1 and AQP-1, kidney sections were first incubated with anti-CTR1 and then with anti-AQP-1 primary antibodies, then they were incubated with the mixture of secondary antibodies.

### 4.6. Statistical Analysis

Data were analyzed for normal distribution using the Shapiro–Wilk test. Differences between the two groups were compared by parametric, two-tailed ANOVA tests or non-parametric two-tailed Kruskal–Wallis ANOVA tests combined with proper post hoc tests (Tukey test and Dunn test, respectively). A value of *p* < 0.05 was considered statistically significant.

## Figures and Tables

**Table 1:** Copper concentration in the liver, kidney and urine of wild-type (w-t) and *mosaic* mutant mice measured by atomic absorption spectrophotometry (AAS). Wild-type and *mosaic* mutant mice were treated with subcutaneous injections of 50 μL of 0.01% CuCl_2_ (Sigma) solution every 2 days from day 2 to day 44 after birth. The total amount of copper administrated per animal during this period was 100 μg. Untreated *mosaic* mutant males die at about day 16 after birth but copper therapy reduced the lethal effect of mutation. Abbreviations: ^a^—significantly different from w-t (control group); ^b^—significantly different from *ms/−* (Cu) (CuCl_2_-injected *mosaic* mutants group); ^c^—significantly different from *ms/−* (untreated mosaic mutants group); ^d^—significantly different from w-t (CuCl_2_-injected, wild-type group); *p* < 0.05—*; *p* < 0.01—**; *p* < 0.001—***; *p* < 0.0001—****; N.A.—non-estimated.

Age/Genotype	Copper Concentration [μg/g Wet Tissue]		
	Liver	Kidney	Urine
14-day-old w-t (5)	8.01 ± 2.11	2.06 ± 0.22	0.08 ± 0.03
14-day-old w-t (Cu) (5)	67.40 ± 2644 ^a^ **^, b^ **	3.13 ± 1.21 ^b^ ****	0.03 ± 0.02
14-day-old *ms/−* (5)	2.49 ± 0.26 ^a^ **^, b^ **	6.31 ± 1.36 ^b^ ***^, c^ ***	N.A.
14-day-old *ms/−* (Cu) (5)	3.80 ± 0.58 ^a^ **^, c^ ***^, d^ **	24.58 ± 828 ^a^ ****^, c^ ***^, d^ ****	0.29 ± 0.13 ^a^ *^, d^ *
45-day-old w-t (5)	5.31 ± 0.98	3.42 ± 0.61	0.21 ± 0.13
45-day-old w-t (Cu) (5)	4.45 ± 1.26	3.81 ± 0.39 ^b^ ****	0.16 ± 0.07
45-day-old *ms/−* (Cu) (5)	3.41 ± 0.52 ^a^ *	4051 ± 10.12 ^a^ ****^, d^ ****	0.23 ± 0.193
6-month-old w-t (5)	4.17 ± 0.84	4.40 ± 1.00	0.15 ± 0.05
6-month-old w-t (Cu) (5)	4.65 ± 0.69	3.70 ± 0.60 ^b^ ****	0.19 ± 0.11
6-month-old *ms/−* (Cu) (5)	3.44 ± 0.34 ^a^ *	22.70 ± 5.20 ^a^ ****^, d^ ****	0.17 ± 0.07

## Data Availability

Not applicable.
